# Microstructural, Thermoelectric and Mechanical Properties of Cu Substituted NaCo_2_O_4_

**DOI:** 10.3390/ma15134470

**Published:** 2022-06-24

**Authors:** Sanja Perać, Slavica M. Savić, Zorica Branković, Slavko Bernik, Aleksandar Radojković, Sanja Kojić, Dragana Vasiljević, Goran Branković

**Affiliations:** 1 Institute for Multidisciplinary Research, University of Belgrade, Kneza Višeslava 1, 11030 Belgrade, Serbia; zorica.brankovic@imsi.bg.ac.rs (Z.B.); aleksandarrr@imsi.bg.ac.rs (A.R.); goran.brankovic@imsi.bg.ac.rs (G.B.); 2 Center for Sensing Technologies, Biosense Institute, Dr Zorana Đinđića 1, 21000 Novi Sad, Serbia; slavicas@biosense.rs; 3 Department for Nanostructured Materials, Jožef Stefan Institute, Jamova Cesta 39, 1000 Ljubljana, Slovenia; slavko.bernik@ijs.si; 4 Faculty of Technical Sciences, University of Novi Sad, Trg Dositeja Obradovića 6, 21000 Novi Sad, Serbia; sanjakojic@uns.ac.rs (S.K.); vdragana@uns.ac.rs (D.V.)

**Keywords:** thermoelectrics, microstructure, electrical resistivity, thermal conductivity, mechanical properties

## Abstract

Polycrystalline samples of NaCo_2−x_Cu_x_O_4_ (x = 0, 0.01, 0.03, 0.05) were obtained from powder precursors synthesized by a mechanochemically assisted solid-state reaction method (MASSR) and a citric acid complex method (CAC). Ceramic samples were prepared by pressing into disc-shaped pellets and subsequently sintering at 880 °C in an argon atmosphere. Effects of low concentrations of Cu doping and the above-mentioned synthesis procedures on the thermoelectric and mechanical properties were observed. The electrical resistivity (*ρ*), the thermal conductivity (*κ*) and the Seebeck coefficient (*S*) were measured simultaneously in the temperature gradient (Δ*T*) between the hot and cold side of the sample, and the figure of merit (*ZT*) was subsequently calculated. The *ZT* of the CAC samples was higher compared with the MASSR samples. The highest *ZT* value of 0.061 at Δ*T* = 473 K was obtained for the sample with 5 mol% of Cu prepared by the CAC method. The CAC samples showed better mechanical properties compared to the MASSR samples due to the higher hardness of the CAC samples which is a consequence of homogeneous microstructure and higher density obtained during sintering of these samples. The results confirmed that, besides the concentration of Cu, the synthesis procedure considerably affected the thermoelectric and mechanical properties of NaCo_2_O_4_ (NCO) ceramics.

## 1. Introduction

As the consumption of electrical energy in the world increases and fossil fuel supply decreases, there is a need for new, sustainable energy sources and different technologies in energy conversion. One method is based on thermoelectric (TE) effects and it implies direct conversion of waste heat into electric energy using TE moduli.

The efficiency of thermoelectric materials is determined by the dimensionless figure of merit, *ZT*:

(1) $$ZT=\frac{S^{2}T}{\rho \kappa }$$

where *S*, *T*, *ρ*, *κ* are the Seebeck coefficient (thermopower), temperature, electrical resistivity and thermal conductivity, respectively [1]. Total thermal conductivity consists of electron (*κ_e_*) and phonon parts (*κ_ph_*). A good TE material with high *ZT* also requires high *S*, low *ρ* and low *κ*. In semiconductors, *S* and *ρ* are inversely proportional, so it is difficult to control them simultaneously. Various attempts have been made to improve the figure of merit of TE materials: by doping [2], decreasing of *κ_ph_* by introducing nanoscale preparation techniques [3] or choosing a material with atoms that induce anharmonic vibrations of chemical bonds [4]. Moreover, it can be achieved in layered oxides since their intrinsic crystal structure enables phonon scattering [5]. High *S* together with low *ρ* in Na_x_Co_2_O_4_, as its essential properties, makes it a promising p-type material for potential TE application. The other advantage of this material is that it is non-toxic with high thermal and chemical stability unlike common thermoelectric materials such as Bi_2_Te_3_, Sb_2_Te_3_ and PbTe, which need to be protected from surface oxidation at high temperatures.

NaCo_2_O_4_ belongs to the alkali ternary oxide group, A_x_MO_2_ (A = Na, K; M = Cr, Mn, Co, etc.), with a hexagonal layered structure (*P*63/*mmc* space group symmetry) [6]. It consists of conductive edge–sharing 2D triangle CoO_2_ sheets and insulating Na layers, alternately stacked along the *c*-axis [6,7]. Electrons in the CoO_2_ layer are localized due to the strong electron correlation, while Na layers serve as regions for phonon scattering. Each of these layers has its function which can be independently controlled [6]. There are three types of the crystal structure of sodium cobaltite, depending on the sodium stoichiometry: P2, γ-Na_x_Co_2_O_4_ (1.0 ≤ x ≤ 1.4), P3, δ-Na_x_Co_2_O_4_ (1.1 ≤ x ≤ 1.2) and O3, α-Na_x_Co_2_O_4_ (1.8 ≤ x ≤ 2.0) [8]. The best thermoelectric performance shows the P2 structure [9].

The thermoelectric properties of polycrystalline NaCo_2_O_4_ can be further improved by introducing dopants in place of Na or Co. Na-site doping with high valence ions decreases carrier density [10], while Co-site doping affects physical properties through changes in the band structure and transport mechanism [11]. Cu-doping of Na_x_Co_2_O_4_ was previously reported by Park and coworkers [12], but secondary phases were present in all samples. Thus, one of the main challenges in obtaining pure NCO is the synthesis process and Na evaporation, which changes the stoichiometry of the obtained ceramic [13]. Regarding the practical application of NCO in TE generators at high temperatures, it is necessary to investigate its thermoelectric properties at high temperatures and mechanical properties in general. Some authors investigated the influence of different weight% of Ag on fractural strength of Ca_3_Co_4_O_9_ samples [14]. They reported that Ag filled the intergranular holes and drastically enhanced mechanical properties, providing plastic regions which prevented crack spreading [14]. Unlike structural, microstructural and thermoelectric properties of sodium cobaltite, which were the subject of many investigations [12,15,16], there are scarce data about its mechanical properties.

The aim of this work was to investigate the influence of low dopant concentrations and different syntheses methods on thermoelectric and mechanical properties of ceramic NaCo_2−x_Cu_x_O_4_ (x = 0, 0.01, 0.03, 0.05). To reduce synthesis time to obtain polycrystalline samples, lower sintering temperature, prevent Na evaporation and improve mixing of the precursors, a citric acid complex method was applied. Alternatively, a mechanochemically assisted solid-state reaction method was used as another approach for comparison. The material’s mechanical behavior and its potential for construction of TE modulus were estimated. For that purpose indentation testing was carried out to determine the hardness and elastic modulus of materials, and force-displacement curves were recorded. In this way, high-temperature thermoelectric and mechanical properties reported in this work, together with already published low-temperature TE performance [13], provide a more complete understanding of Cu-doped NaCo_2_O_4_ behavior.

## 2. Materials and Methods

Polycrystalline samples of NaCo_2-x_Cu_x_O_4_ (x = 0, 0.01, 0.03 and 0.05) were prepared with a mechanochemically assisted solid-state reaction method (MASSR) and a citric acid complex method (CAC). The MASSR precursors were prepared using Na_2_CO_3_, Co_3_O_4_ and CuO, all manufactured by Sigma Aldrich. An excess of Na_2_CO_3_ was added to replace sodium evaporation at high temperatures [13]. Appropriate amounts of powders were mechanically activated in a planetary ball mill (Fritsch pulverisette 5) for 3 h. The CAC precursor samples were prepared using an aqueous solution of Na, Co and Cu acetates and citric acid as a chelating agent. An excess of sodium acetate was added to compensate for the evaporation of Na and the molar ratio of citric acid to metal ion was 3:1. Each aqueous solution was gradually heated up to 140 °C under constant stirring to obtain gel and then further heated at 450 °C for 2 h when the dark mass precursor was formed. The CAC powders were finally calcined at 800 °C for 20 h. Preparation of ceramic samples from precursor powders by MASSR and CAC method is presented in Table 1.

Phase identification of the sintered samples was performed using X-ray powder diffraction (XRD, Rigaku RINT 2000) with Fe Kα radiation as an X-ray source. The microstructure of the sintered samples was analyzed by TESCAN Vega TS 5130 MM scanning electron microscope (SEM) equipped with a back-scatter electron (BSE) detector. The Seebeck coefficient (*S*), the electrical resistivity (*ρ*) and the thermal conductivity (*κ*) were measured simultaneously on a cylindrical sample with a diameter and height of about 10 mm, under vacuum of 10**^−^**^2^–10**^−^**^3^ Pa using Z-meter, based on the “large Δ*Τ* method” [17]. The sample was heated on one side to a set temperature of 830 K. The thermoelectric parameters (*ρ*, *S*, and ***κ***) were measured simultaneously during cooling of the sample in a time interval of one minute until the temperature on the hot side of the samples dropped to 320 K. Accordingly, the thermoelectric parameters were determined for a temperature gradient that is established between the hot and cold sides of the sample at the time of each measurement; thus obtained values therefore represent actual thermoelectric response of a material under conditions of application. Mechanical properties were investigated by determining the hardness and the Young’s modulus of elasticity using Agilent Nanoindenter G200. The surface approach velocity of the indenter (Berkovich pyramid) and the Poisson ratio were set to 10 nm/s and 0.25, respectively.

## 3. Results

**Figure 1:**
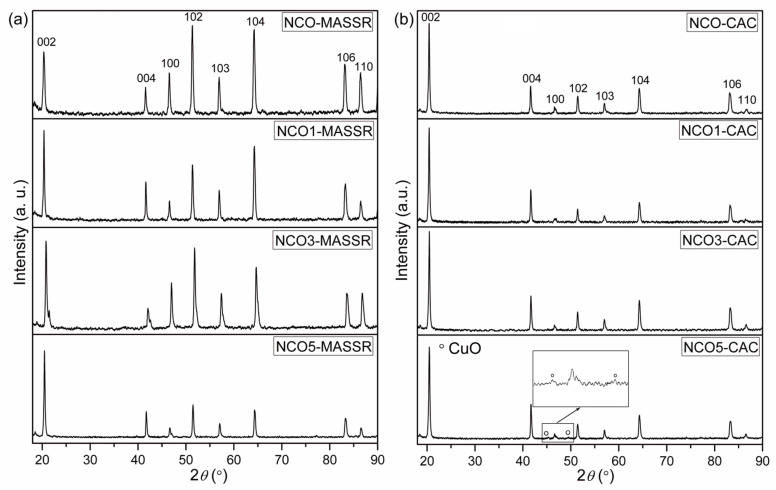
XRD patterns of the (**a**) MASSR and (**b**) CAC samples.

The X-ray powder diffraction patterns of the sintered samples are displayed in Figure 1. MASSR ceramic samples (Figure 1a) contained only the diffraction peaks indexed to the single-phase γ-NaCo_2_O_4_ (JCPDF card No. 73-0133, space group *P*63/*mmc*). On the other hand, the CAC ceramic sample with x = 0.05 (Figure 1b) showed two reflections at approximately 45° and 49° 2 *θ* originating from the secondary phase CuO (JCPDF card No. 89-2529 and space group *C*2/*c*).

The lattice parameters *a* and *c* of both types of the samples were calculated according to the XRD patterns in the computer programme LSUCRI, as was explained in detail in our previous work [13]. The parameters *a* and *c* are similar to the standard values of *a* = 2.843 Å and *c* = 10.881 Å, reported in JCPDF card No. 73-0133 [8]. Both parameters were higher for the MASSR than for the CAC samples for the same Cu amount. This was the result of higher concentration of defects (dislocations and point defects) in the crystal lattice of the MASSR samples, induced by mechanical activation of the solids [18].

**Figure 2:**
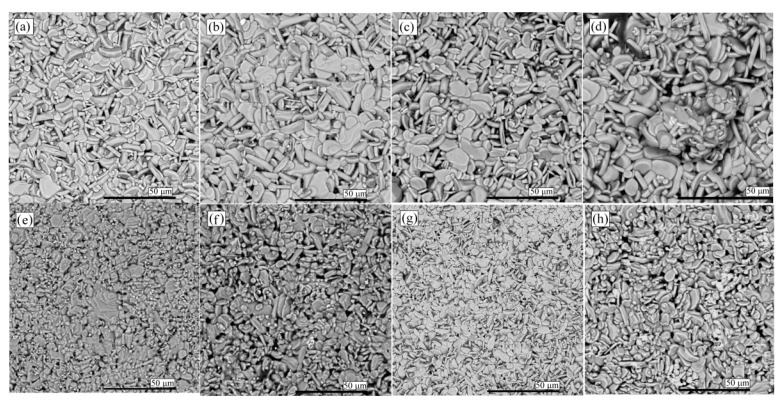
SEM micrographs (BSE mode) from the surface of (**a**) NCO-MASSR, (**b**) NCO1-MASSR, (**c**) NCO3-MASSR, (**d**) NCO5-MASSR, (**e**) NCO-CAC, (**f**) NCO1-CAC, (**g**) NCO3-CAC and (**h**) NCO5-CAC.

SEM micrographs (Figure 2) of both types of samples showed uniformed microstructure with layered, plate-like grains, which increased with increasing Cu concentration. MASSR grains were larger than CAC grains, with lengths ranging from 8–13 μm for the MASSR and 4 up to 11 μm for the CAC samples. The densities of the MASSR samples were between 88% and 91% of the theoretical value, and approximately 92–93% for the CAC samples. The lowest density (88%) was calculated for NCO5-CAC. Moreover, the SEM analysis indicated the highest porosity of this sample. In samples NCO5-MASSR and NCO5-CAC, white precipitates were noticed and EDX analysis confirmed a higher Cu amount in these spots, although secondary phases were not detected in the XRD of the NCO5-MASSR sample [13].

The electrical resistivity, the thermal conductivity and the Seebeck coefficient of all samples were measured in the temperature range 320–830 K. This is a sequel of our previous research, where the thermoelectric properties were presented in low temperature regions, below room temperature [13]. Since the thermoelectric parameters (i.e., *ρ*, *S*, *κ* and *ZT*) were measured by the large Δ*T* method and thus determined in the temperature gradient, their values are graphically represented according to the temperature on the hot side of the sample and the temperature difference between the hot and cold sides of the sample at this temperature of hot side.

**Figure 3:**
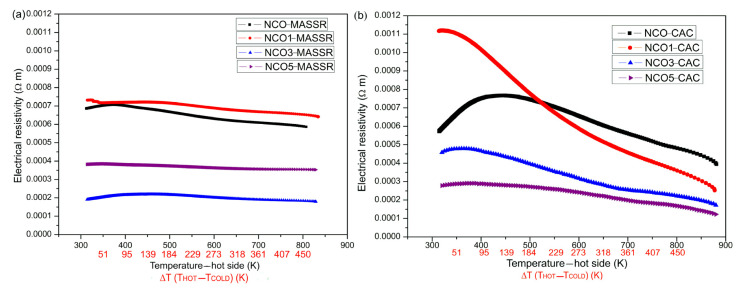
The electric resistivity of the (**a**) MASSR and (**b**) CAC samples for the temperature gradients Δ*T* at the temperatures of the hot side of the samples.

The electrical resistivity of NaCo_2−x_Cu_x_O_4_ (x = 0, 0.01, 0.03 and 0.05) is shown in Figure 3.

In the measured temperature range, measured *ρ* of all samples implied a metal-insulator transition. Metallic behavior of NaCo_2_O_4_ originates from low spin Co^3+^ and Co^4+^ ions. Namely, it is assumed that in stoichiometric NaCo_2_O_4_ there are equal amounts of Co^4+^ and Co^3+^ ions. These ions can occupy low spin state, with electronic configurations, t_2g_^5^ (t_2g_^6^); intermediate, t_2g_^4^e_g_^1^ (t_2g_^5^e_g_^1^); or high spin state, t_2g_^3^e_g_^2^ (t_2g_^4^e_g_^2^). It is assumed that Co ions occupy low spin state. As a result, the t_2g_ band splits into a_1g_ and e’_g_ bands. The e_g_ bands of all CoO_6_ octahedra, regardless of whether they contain Co^4+^ or Co^3+^ ions, overlap and form wide e’_g_-block bands, responsible for metallicity [19,20]. The CAC samples showed smaller resistivity, and among them, the lowest value was obtained for NCO5-CAC. The resistivities of the samples did not follow monotonous behavior of dependencies. This can be attributed to a complex influence of dopant concentration on variety of material properties, such as structure (symetry), microstructure (porosity), phase purity (NCO5-CAC contained traces of CuO) and even Co^3+^/Co^4+^ ratio. Some of these properties can have the opposite effect on the resistivity or thermal conductivity of the samples. Furthermore, different behavior between CAC and MASSR samples can be explained by difference in their microstructure and chemical homogeneity. Larger and flatter grains observed in MASSR samples make them more prone to the anisotropy of the electrical conductivity. Similar behavior was observed by Seetawan et al. [21] and Terasaki et al. [2], where the resistivity curves of NCO samples also showed non-monotonous dependence with the concentration of Ag and Cu, respectively. In general, the electrical conductivity of Na_x_Co_2_O_4_-based systems consists of an ionic and electronic component. The ionic part originates from Na^+^-ions and it is several orders of magnitude lower than the electronic, so the measured conductivity has an electronic character [12]. In the temperature range between 2 and 300 K, CAC samples showed metallic behaviour [13]. Therefore, the mechanism of the electrical conductivity can be explained in the following way. The valence band (VB) and conductive band (CB) consist of Na 3s, Co 3d and O 2p orbitals and transition between VB and CB occurs because of the maximum contribution of 2p and 3d states in VB and CB [22]. Low spin Co^3+^ (3d^6^) and Co^4+^ (3d^5^) ions formed partially filled 3d bands in the CoO_2_ sheets. Overlapping 3d orbital from Co^3+^ and/or Co^4+^-ions and 2p orbital from O^2−^-ion created weak hybridization between these orbitals, and formed a broad Co-O-Co band, located between the valent and conduction band. The transition between valence and conduction band occurred through the Co-O-Co band and in low temperature regions this mechanism of conduction dominated. As the temperature increased, the carriers from Co-O-Co passed in the conduction band; their number also increased with increasing temperature and caused a moderate decrease of the resistivity. Doping with Cu^2+^ in place of Co^3+^/^4+^ increases the number of oxygen vacancies; this can be expressed according to the following equation with Kröger–Vink notation:

(2) $${\mathrm{Co}}^{3+}:\;2Co_{Co}^{x}+O_{O}^{x}+2CuO_{\left(s\right)}\to 2Cu_{Co}^{\prime }+V_{O}^{••}+Co_{2}O_{3\left(s\right)}\;{\mathrm{Co}}^{4+}:\;Co_{Co}^{x}+O_{O}^{x}+CuO_{\left(s\right)}\;\to \;Cu_{Co}^{\prime \prime }+V_{O}^{••}+CoO_{2\left(s\right)}$$

With respect to these equations, the conductivity of the samples should increase with Cu concentration. However, since the other part of the conductivity contribution is related to Co^3+^/Co^4+^ distribution as the intrinsic source of charge carriers, it can be assumed that some charge carrier recombination takes place and reflects on the resistivity trend.

**Figure 4:**
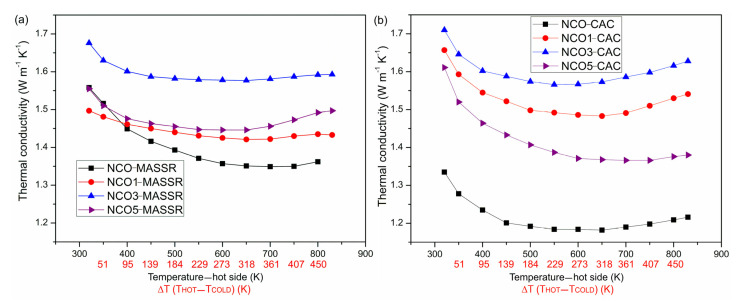
Temperature dependence of the thermal conductivity of the (**a**) MASSR and (**b**) CAC samples for the temperature gradients Δ*T* at the temperatures of the hot side of the samples.

The thermal conductivity of the samples is presented in Figure 4. In this range, *κ* has a parabolic shape. Minimum values for the MASSR samples were between 1.35 W/m K and 1.57 W/m K, and for the CAC samples between 1.18 W/m K and 1.58 W/m K, indicating that preparation methods had a weak influence on *κ*. In both cases, maximum *κ* was obtained for the samples containing 3 mol% of Cu, and the minimum *κ* was obtained for undoped samples. Lower thermal conductivity of the CAC samples is the consequence of enhanced phonon scattering, due to the fine microstructure of the samples, which enables precursor preparation by CAC [7]. The NCO1-MASSR sample possesses smaller grains with irregular forms compared to NCO5-MASSR, which may reflect on its lower thermal conductivity.

**Figure 5:**
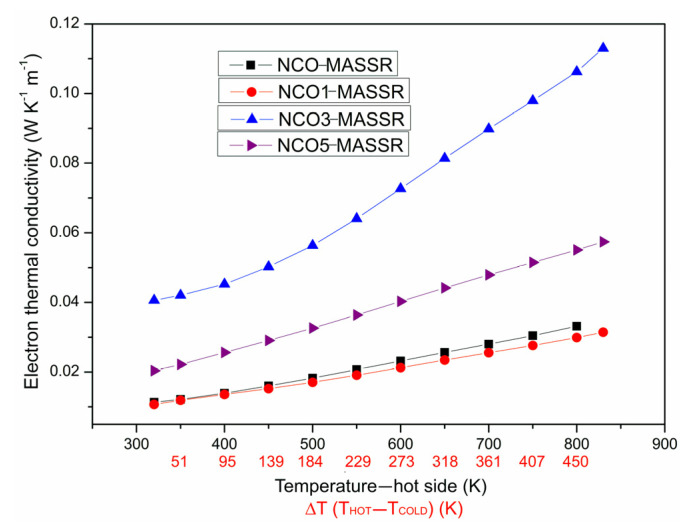
Electron thermal conductivity of MASSR samples for the temperature gradients Δ*T* at the temperatures of the hot side of the samples.

The electron thermal conductivity can be calculated with the Wiedemann–Franz law:

(3) $$\kappa_{el}=\frac{L_{0}T}{\rho }$$

where *L*_0_ is the Lorenz number ($L_{0}=\frac{\pi^{2}k_{B}^{2}}{3e^{2}}$). Its values were one order of magnitude lower than total thermal conductivity (Figure 5), so the measured *κ* came from the lattice [5].

**Figure 6:**
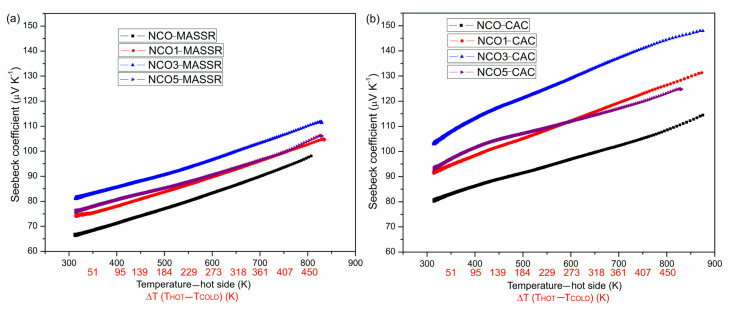
Temperature dependence of the Seebeck coefficient of the (**a**) MASSR and (**b**) CAC samples for the temperature gradients Δ*T* at the temperatures of the hot side of the samples.

The Seebeck coefficient was positive in the whole temperature range (Figure 6), indicating that major conductivity carriers were holes and increased with increasing temperature [12].

The CAC samples showed larger values compared with the MASSR and in both cases the doped samples showed higher *S* compared with the undoped ones. The highest value was found in samples with 3 mol% of Cu (145 μV/K for NCO3-CAC and 110 μV/K for NCO3-MASSR). High *S* was the consequence of the high electron correlation present in this type of compound [2,5]. As we have already emphasized, the CAC method enabled better homogenization of the constituents during the synthesis, resulting in homogeneous precursor powders, higher density and smaller grains of the sintered ceramics. Bearing in mind the above properties, better results of TE measurements were confirmed in the CAC samples. Moreover, *S* of undoped samples (111 μV/K for NCO-CAC and 100 μV/K for NCO-MASSR at T_HOT_ = 830 K, Δ*T* = 473 K) was comparable with already reported values [21,23,24,25].

**Figure 7:**
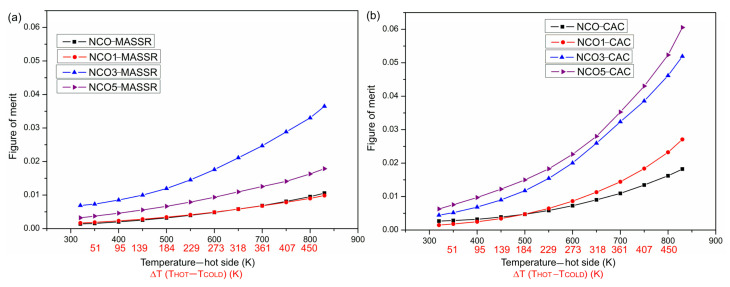
Temperature dependence of the figure of merit of the (**a**) MASSR and (**b**) CAC samples for the temperature gradients Δ*T* at the temperatures of the hot side of the samples.

The figure of merit was calculated based on the obtained values for the electrical resistivity, the Seebeck coefficient and the thermal conductivity and its temperature dependence are presented in Figure 7. 

Taking into account both synthesis methods, the highest *ZT* was obtained for the sample NCO5-CAC (0.061 at Δ*T* = 473 K), mainly due to high *S* and low *ρ*, which is in accordance with *ZT* = 0.07, obtained for Na_0.75_CoO_2_ epitaxial film [25]. This value was more than three times larger than *ZT* for the undoped CAC sample and 1.7 times larger than the highest value obtained for the MASSR sample (*ZT*(NCO3-MASSR) = 0.036 at Δ*T* = 473 K). Although the sample NCO3-MASSR showed increased *κ*, it also showed enhanced *S*, which is a more dominant characteristic because of squared *S* in the equation for *ZT*. The difference between the minimum and maximum *κ* for MASSR and CAC samples did not have much of an impact on the final result of the figure of merit.

Within mechanical properties, Young’s modulus of elasticity and hardness of both types of samples were determined. The technique involved the recording of force and displacement when an indentation was made. While nanoindentation tests were performed, the indentation tip was pressed on the surface of the sample and load-displacement curves were recorded. The average values of the mechanical properties obtained for NaCo_2−x_Cu_x_O_4_ (x = 0, 0.01, 0.03 and 0.05) ceramics are given in Table 2.

**Figure 8:**
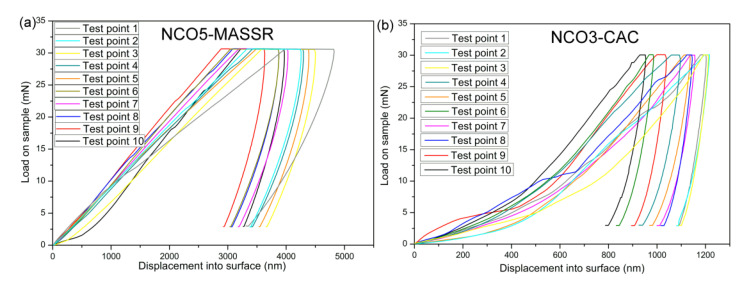
Load-displacement curves for (**a**) NCO5-MASSR and (**b**) NCO3-CAC samples.

**Figure 9:**
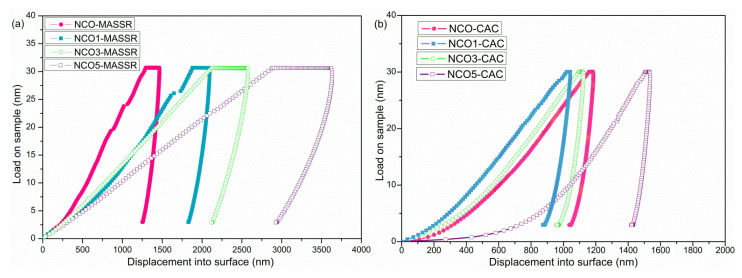
Load-displacement curves obtained during nanoindentation test of (**a**) MASSR and (**b**) CAC ceramics.

The hardness of the CAC samples was higher compared to the MASSR samples and it could be related to the homogeneous microstructure, higher density of these samples and higher concentration of microstructural defects in the MASSR samples. The NCO5-MASSR sample possesses the lowest density and the most porous microstructure, and therefore a significant reduction of the Young’s modulus and the hardness was obtained for this sample. Moreover, the modulus and the hardness of the NCO1-CAC sample, which were estimated to be 65.2 GPa and 1.41 GPa, respectively, were almost 2 and 1.5 times, respectively, larger than the one reported for Bi_2_Te_3_, common-type TE material [26]. The reason for increased hardness can be explained by strong cohesion forces and increased number of grain boundaries, which hinder crack propagation [27].Furthermore, the absence of secondary phases, which can hinder crack propagation, can be attributed to enhanced hardness. Uniform values of the Young’s modulus for CAC samples reflected on the properties of bulk material and highlighted the advantage of the citric acid method for obtaining homogeneous microstructures. Load-displacement curves for the MASSR and CAC ceramic samples are presented in Figure 8 and Figure 9. Different shapes of the curves for the same sample indicated some inhomogeneity of the sample (Figure 8). Smaller deviations of the curves for CAC samples (Figure 9) pointed to a higher degree of homogeneity. Lower depths of impression came as a result of increased hardness. Taking into account obtained results, the highest figure of merit and the best mechanical properties exhibited the NCO3-CAC sample, and can be considered for practical application.

Having in mind all the results presented in this work, it is apparent that the synthesis procedure notably affects the phase purity, microstructure, thermoelectric and also mechanical properties of NCO ceramics.

## 4. Conclusions

Polycrystalline samples of NaCo_2−x_Cu_x_O_4_ (x = 0, 0.01, 0.03, 0.05) were synthesized by a mechanochemically assisted solid-state reaction and a citric acid complex method followed by different heat treatments. The thermoelectric parameters were determined by the large Δ*T* method; their values therefore indicate actual thermoelectric response of material in temperature gradients between the hot and cold sides, directly relevant to their applications. The shape of the curve *ρ* = f(*T*) for all samples implied metal-insulator transition. Minimum thermal conductivity was obtained for the undoped samples in both cases, and the difference between the minimum and maximum of 15–20% did not significantly affect the final result. The Seebeck coefficient was positive, increased with temperature and it was enhanced for all Cu-substituted samples. The highest *ZT* of 0.061 at Δ*T* = 473 K was obtained for the sample with 5 mol% of Cu and it was 1.7 times larger than the maximum value obtained for the MASSR sample. The CAC samples showed increased hardness due to the homogeneous microstructure and higher density compared with the MASSR samples. Smaller deviations of the load-displacement curves for the CAC samples were the result of the higher degree of homogeneity. In general, the CAC method enabled a better homogenization of the constituents during the synthesis, obtaining homogeneous precursor powders and smaller grains of the sintered ceramics, and thus the expected better results of TE and mechanic measurements were confirmed.

## Figures and Tables

**Table 1:** Preparation of ceramic samples from precursor powders by MASSR and CAC method.

mol% of Cu	MASSR Method			CAC Method		
	Denotation of Samples	Pressing Pressure	Sintering Conditions	Denotation of Samples	Pressing Pressure	Sintering Conditions
0	NCO-MASSR	590 MPa	880 °C for 24 h	NCO-CAC	390 MPa	880 °C for 20 h
1	NCO1-MASSR			NCO1-CAC		
3	NCO3-MASSR			NCO3-CAC		
5	NCO5-MASSR			NCO5-CAC		

**Table 2:** Mechanical properties of MASSR and CAC ceramics determined by the nanoindentation.

mol% Cu	MASSR		CAC	
	Modulus at Max Load (GPa)	Hardness at Max Load (GPa)	Modulus at Max Load (GPa)	Hardness at Max Load (GPa)
0	30.2	0.63	63.6	0.97
1	22.2	0.49	65.2	1.41
3	8.2	0.26	64.1	1.15
5	2.0	0.03	61.8	0.63

## References

[1] Bhandari C.M., Rowe D.M. (1995). CRC Handbook of Thermoelectrics.

[2] Terasaki I. Anomalous Co-site substitution effects on the physical properties of the thermoelectric oxide NaCo_2_O_4_. Proceedings of the 19th International Conference on Thermoelectrics (ICT 2000).

[3] Sevinçli H., Sevik C., Çağin T., Cuniberti G. (2013). A bottom-up route to enhance thermoelectric figures of merit in graphene nanoribbons. Sci. Rep..

[4] Heremans J.P. (2014). The ugly duckling. Nature.

[5] Takahata K., Iguchi Y., Tanaka D., Ito T., Terasaki I. (2000). Low thermal conductivity of the layered oxide (Na, Ca) Co_2_O_4_: Another example of a phonon glass and an electron crystal. Phys. Rev. B.

[6] Tang X., Tritt T.M. (2008). Overview of thermoelectric sodium cobaltite: Na_x_Co_2_O_4_. J. South Carol. Acad. Sci..

[7] Ito M., Nagira T., Furumoto D., Katsuyama S., Nagai H. (2003). Synthesis of Na_x_Co_2_O_4_ thermoelectric oxides by the polymerized complex method. Scr. Mater..

[8] Fouassier C., Matejka G., Reau J.-M., Hagenmuller P. (1973). Sur de nouveaux bronzes oxygénés de formule Na_x_CoO_2_ (x ≤ 1). Le systéme cobalt-oxygéne-sodium. J. Solid State Chem..

[9] Rowe D.M. (2006). Thermoelectrics Handbook: Macro to Nano.

[10] Kawata T., Iguchi Y., Itoh T., Takahata K., Terasaki I. (1999). Na-site substitution effects on the thermoelectric properties of NaCo_2_O_4_. Phys. Rev. B.

[11] Asahi R., Sugiyama J., Tani T. (2002). Electronic structure of misfit-layered calcium cobaltite. Phys. Rev. B.

[12] Park K., Jang K.U., Kwon H.-C., Kim J.-G., Cho W.-S. (2006). Influence of partial substitution of Cu for Co on the thermoelectric properties of NaCo_2_O_4_. J. Alloy. Compd..

[13] Pršić S., Savić S.M., Branković Z., Vrtnik S., Dapčević A., Branković G. (2015). Mechanochemically assisted solid-state and citric acid complex syntheses of Cu-doped sodium cobaltite ceramics. J. Alloy. Compd..

[14] Kahraman F., Madre M.A., Rasekh Sh., Salvador C., Bosque P., Torres M.A., Diez J.C., Sotelo A. (2015). Enhancement of mechanical and thermoelectric properties of Ca_3_Co_4_O_9_ by Ag addition. J. Eur. Ceram. Soc..

[15] Tian Z., Wang X., Liu J., Lin Z., Hu Y., Wu Y., Han C., Hu Z. (2016). Power factor enhancement induced by Bi and Mn co-substitution in Na_x_CoO_2_ thermoelectric materials. J. Alloy. Compd..

[16] Tsai P.H., Zhang T.S., Donelson R., Tan T.T., Li S. (2011). Power factor enhancement in Zn-doped Na_0.8_CoO_2_. J. Alloy. Compd..

[17] Presečnik M., de Boor J., Bernik S. (2016). Synthesis of single-phase Ca_3_Co_4_O_9_ ceramics and their processing for a microstructure-enhanced thermoelectric performance. Ceram. Int..

[18] Fernández-Bertran J.F. (1999). Mechanochemistry: An overview. Pure Appl. Chem..

[19] Raveau B. (2005). Transition metal oxides: Promising functional materials. J. Eur. Ceram. Soc..

[20] Whangbo M.-H., Dai D., Kremer R.K. (2006). On the Origin of the Metallic and Anisotropic Magnetic Properties of NaxCoO_2_ (x ≈ 0.75). Inorg. Chem..

[21] Seetawan T., Amornkitbamrung V., Burinprakhon T., Maensiri S., Kurosaki K., Muta H., Uno M., Yamanaka S. (2006). Thermoelectric properties of Na_x_Co_2_O_4_/Ag composites. J. Alloy. Compd..

[22] Akter T., Gafur A., Razzaque Sarker A. (2014). Sodium nonstoichiometry effects on the crystal structure, electronic, optical and thermoelectric properties of sodium cobaltite. Int. J. Innov. Res. Adv. Eng..

[23] Zhu P., Takeuchi T., Ohta H., Seo W.-S., Koumoto K. (2005). Preparation and thermoelectric properties of Na_x_CoO_2_/Co_3_O_4_ layered nano-composite. Mater. Trans..

[24] Tajima S., Tani T., Isobe S., Koumoto K. (2001). Thermoelectric properties of highly textured NaCo_2_O_4_ ceramics processed by the reactive templated grain growth (RTGG) method. Mater. Sci. Eng. B-Adv..

[25] Takashima Y., Zhang Y.-Q., Wei J., Feng B., Ikuhara Y., Cho H.J., Ohta H. (2021). Layered cobalt oxide epitaxial films exhibiting thermoelectric ZT = 0.11 at room temperature. J. Mater. Chem. A.

[26] Zhao L.-D., Zhang B.-P., Li J.-F., Zhou M., Liu W.-S., Liu J. (2008). Thermoelectric and mechanical properties of nano-SiC-dispersed Bi_2_Te_3_ fabricated by mechanical alloying and spark plasma sintering. J. Alloy. Compd..

[27] Guillemet-Fritsch S., Salmi J., Sarrias J., Rousset A., Schuurman S., Lannoo A. (2004). Mechanical properties of nickel manganites-based ceramics used as negative temperature coefficient thermistors (NTC). Mater. Res. Bull..

